# Effects of a 1 year development programme for recently graduated veterinary professionals on personal and job resources: a combined quantitative and qualitative approach

**DOI:** 10.1186/s12917-015-0627-y

**Published:** 2015-12-30

**Authors:** N. J. J. M. Mastenbroek, P. van Beukelen, E. Demerouti, A. J. J. A. Scherpbier, A. D. C. Jaarsma

**Affiliations:** Chair Quality Improvement in Veterinary Education, Faculty of Veterinary Medicine, Utrecht University, P.O.Box 80163, 3508TD Utrecht, The Netherlands; Industrial Engineering and Innovation Sciences, Eindhoven University of Technology, Eindhoven, The Netherlands; Education Institute, Faculty of Health, Medicine and Life Sciences, Maastricht University, Maastricht, The Netherlands; Center for Research and Innovation in Medical Education, University Medical Center Groningen, Groningen, The Netherlands

**Keywords:** Burnout, Resources development programme, JD-R model, Job resources, Peer group coaching, Personal resources, Transition, Veterinary, Work engagement

## Abstract

**Background:**

The early years in professional practice are for many veterinary and medical professionals a period of great challenges and consequently increased stress levels. Personal resources appear to have a positive impact on the course of this transition period. Personal resources are defined as developable systems of positive beliefs about one’s self and the world that are generally linked to resilience. They are negatively related to burnout and positively and reciprocally to job resources, work engagement and job performance. With the aim of enhancing personal resources of recently graduated veterinarians, a 1 year multi-modular resources development programme was designed. This study was conducted to analyse:

1. if and how the development programme affected participants’ personal resources, and
2. if and how personal resources affected participants’ work characteristics and work engagement.

**Results:**

ᅟ

Quantitative study: Twenty-five participants and ten non-participants completed an online survey covering personal resources, job resources and work engagement at the start and finish of the programme. Results showed a significant increase of personal resources in participants for self-reported ratings of proactive behaviour (Effect Size =−0.4), self-efficacy (Effect Size =−0.6) and reflective behaviour (Effect Size =−0.6). Results of the control group were not significant, although some moderate effect sizes were found.

Qualitative study: Additionally 16 semi-structured interviews with participants of the programme were taken 6 months after finishing the programme. Analysis of the interviews revealed that participants also developed other important personal resources namely self-acceptance, self-esteem, awareness of own influence and responsibility. The reflection process, which took place in the course of the programme, seemed to be a necessary step for the development of the other personal resources. According to participants of the resources development programme, the increase in personal resources also gave rise to an increase in job resources.

**Conclusion:**

The multi-modular resources development programme seems to support development of participants’ personal resources. Because personal resources are beneficial in improving well-being irrespective of where an individual starts working, it is important to give them explicit attention in educational settings.

**Electronic supplementary material:**

The online version of this article (doi:10.1186/s12917-015-0627-y) contains supplementary material, which is available to authorized users.

## Background

Transition from student to professional (whether veterinarian or medical), from a safe learning environment to a professional environment with great responsibilities, means entering a period of rapid personal and professional development, often characterized by elevated levels of stress [1–9]. Inadequate support, negative experiences, making mistakes and being overworked and underpaid, can make the first year after graduation a very critical period in the career and lives of the recently graduated professional [1, 6, 9]. This applies not only for veterinarians but also for other medical health care professional such as nurses [10–12], residents and general practitioners [13–16]. Although many medical health care professionals, in contrast to veterinarians, enter a structured training programme after graduation from medical school, research in this domain showed that they are struggling with the same problems. Apart from a negative experience due to increased stress levels, the transition period can also be interpreted positively as challenging, full of learning opportunities and opportunities for high performance [5]. In a study among recently graduated veterinary professionals, Mastenbroek et al. [17] investigated work-related well-being and its predictors in the transition period, using the Job Demands-Resources (JD-R) model as a theoretical model [18, 19]. Their results showed that one in seven Dutch veterinarians is likely to be burnt-out within 10 years of graduation, while only one in eight veterinarians qualifies him/herself as highly engaged in that same period. Well-being was predicted by both work-related (job demands and job resources) and person-related (personal resources) predictors [17].

**Fig. 1:**
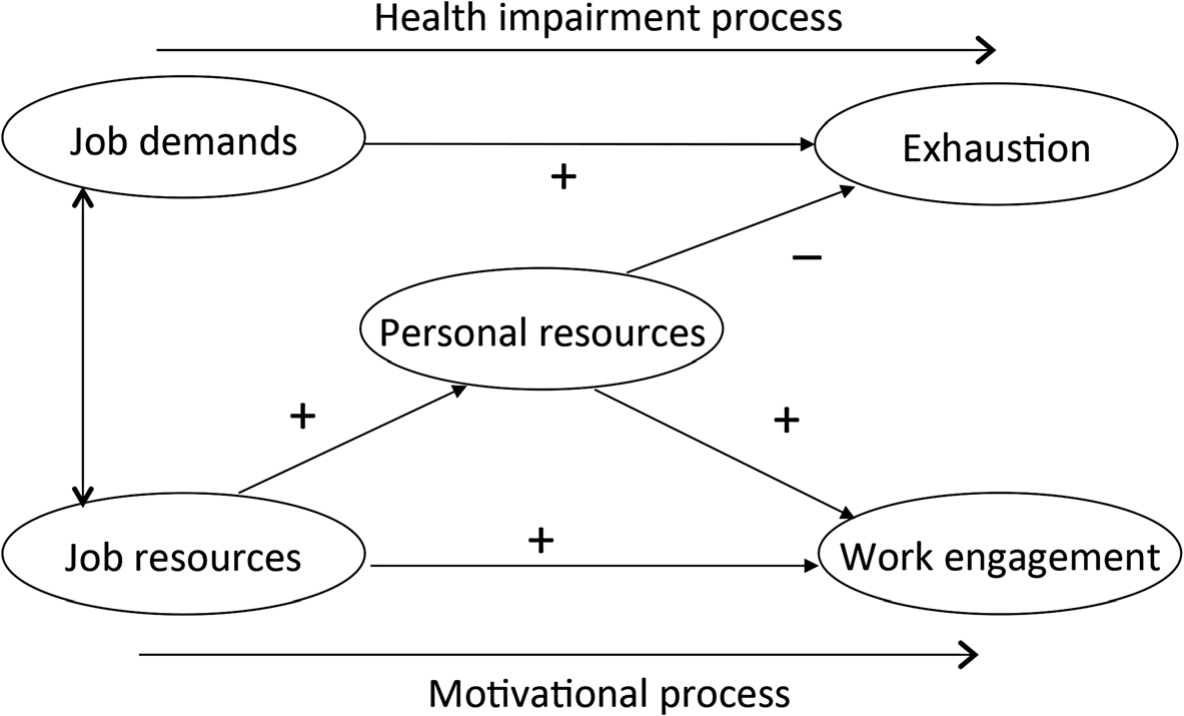
The extended Job Demands–Resources Model [25]

The JD-R model is an occupational stress model that posits two broad categories of work characteristics: job demands and job resources [18, 19]. Job demands are aspects of work that require sustained physical or mental effort on the part of the employee and are thus associated with psycho-physiological costs. Examples are work-home interference, workload, job insecurity and role conflicts. Job resources are aspects of the work that are functional in dealing with high demands and achieving occupational goals. They are also important in their own right as stimulants of personal growth [20]. Examples are, autonomy, feedback from work and support from colleagues and supervisor. According to the JD-R model, job demands can evoke an energy depletion process, potentially leading to a breakdown (or burnout) when individuals fail to recover adequately [21]. Job resources on the other hand, induce a motivational process, which can promote work engagement. In addition to job resources, we can also distinguish personal resources. Personal resources are developable systems of positive beliefs about one’s self and the world that are generally linked to resilience, i.e. people’s sense of being in control and able to influence their environment successfully [22]. This definition encompasses a feeling of being appreciated and in control as well as skills and attitudes that facilitate these feelings. Examples of personal resources are self-esteem, self-efficacy, optimism, and a pro-active attitude [17, 23]. Personal resources are negatively related to burnout and positively and reciprocally to work engagement and job resources [24] and to performance [25]. Figure 1 presents the extended JD-R model (including personal resources) that we validated for the veterinary profession in an earlier study [25]. Job and personal resources have been found particularly beneficial for employees’ work engagement when job demands are high [26]. Because of their positive relationship to mental well-being, these personal resources are suggested to represent a target for interventions aimed at facilitating the transition period and increasing the well-being of young veterinary professionals.

For educators it is important to understand what can contribute to a more positive progress of the transition period [27]. The present study was conducted to analyse the effects of an intervention on personal resources: a 1 year resources development programme was designed for veterinary professionals who had graduated during the last 5 years, focusing on broadening and/or enhancing participants’ personal resources. The following questions were investigated:

1. Are self-reported levels of personal and job resources and work engagement higher at the end of the intervention as opposed to their levels at the start of the intervention?
2. How did the programme affect participant’s personal resources?
3. How did personal resources affect participant’s work and work environment and participant’s work engagement?

## Methods

### Context

In the Netherlands, students may register for veterinary education at the Faculty of Veterinary Medicine after having completed high school. The veterinary education programme lasts for 6 years. After graduation, 75 % of the veterinary professionals (of which 80–85 % females) chooses for a career in veterinary practice. After a few years of working as an employee, they often continue their career in veterinary practice as a self-employed vet. The other 25 % prefer employment in industry, educational institutes, research or a job in the public domain. There is no formal additional training programme for those who enter veterinary practice.

### Programme design

In 2010, the Royal Netherlands Veterinary Association introduced a resources development programme for young veterinary professionals with the aim of enhancing work engagement by broadening and increasing participants’ personal resources. Because the needs of participants are various, the programme was designed in a way that it enabled participants to set their own learning objectives and to work thereto during the trajectory. The modules vary regarding the input they provide for reflection and learning at different levels (i.e. context, behaviour, beliefs or competencies) [28]. In the course of this article, the ‘resources development programme’ will be referred to in abbreviated form as the ‘development programme’.

The development programme consists of:

1. An intake procedure including a talent assessment, collection of 360° feedback at the workplace and an intake meeting that serves to identify goals for improvement and commitment to work on these goals throughout the programme.
2. Various modules with an emphasis on reflection and experimenting with new behaviour. All participants were enrolled in the general programme. Participants met every 6 weeks for 10 months. Altogether, there were six training days. A training day consisted of three parts: a) looking back on the past 6 weeks, b) intervision (peer-coaching) and c) half a day professional skills training. Each training day ended with updating existing learning objectives or with preparing new ones. In the period between two meetings, participants worked on their learning objectives and applied new insights and skills in their work. When necessary the general programme was supplemented with individual coaching and E-learning modules. The programme was facilitated by two professional coaches.^1^

Registration for the programme was open to all veterinary professionals having graduated between 0 and 5 years. Recruitment of participants took place by means of an electronic newsletter from the Royal Netherlands Veterinary Association and through an announcement in the Netherlands Journal of Veterinary Science [29]. There was no active selection of participants. Participation was voluntary and costs were about 2000 euro.

This manuscript reports one study combining a qualitative and a quantitative part including an online survey and semi-structured interviews.

#### Quantitative study

### Procedure

Measurements were taken at two different moments: at the time of the onset of the development programme (Time 1), and 10 months later, when the development programme was completed (Time 2). On the first day of the programme an introduction to the research was held. At this introductory meeting the goal of the study and the entire procedure was explained, addressing the confidentiality of the data. Participants of the programme were asked to volunteer for our study. A day after the meeting, invitations to participate in the research were sent individually by e-mail to all participants of the development programme. The e-mail contained an internet link which directed the participants to an online survey. Two and 4 weeks after the distribution of the questionnaire, reminders were sent by personalized e-mail. Participants who completed the questionnaire at Time 1 received an invitation to complete the survey again at Time 2. The T2 questionnaire differed from the T1 questionnaire with respect to one question that was added to the T2 questionnaire: we asked participants whether anything had changed concerning the organisation they worked for. If participants had changed jobs and worked for another organization, the questions concerning job recourses were skipped, because in that case job resources at T2 and T1 cannot be compared.

### Participants

In total 46 participants (male/female: 9/37) entered the development programme in three groups starting in April 2010, September 2010 and July 2011. All participants completed the full programme. There were no dropouts. The male–female ratio of participants to the development programme was representative for veterinarians having graduated in the last 5 years. Thirty-three participants (response rate (RR) = 72 %) completed the first questionnaire (Time 1). Twenty-five participants (RR = 54 %) completed both questionnaires (Time 1 and Time 2) (male/female: 3/22, mean age: 29 years, mean work experience: 2.8 years, 22 persons were employed in veterinary practices consisting of an average of nine vets, and three were employed in other domains). We asked respondents to fill in the names of two colleagues who did not participate in the development programme (preferably with the same sex, the same year of graduation, the same type of work, but working in another veterinary clinic). These colleagues were approached to participate in the control group which serves as a measure for normal development over time in the first years of the career. The control group consisted of 22 veterinarians who were invited to complete the survey. Eighteen persons (RR = 82 %) completed the first questionnaire. Ten persons (RR = 45 %) completed both questionnaires (male/female: 1/9 mean age: 29 years, mean work experience: 3.1 years, all ten persons were employed in veterinary practices consisting of an average of seven vets).

### Measures

*Personal and job resources* were measured using 35 items of the Veterinary Demands and Resources Questionnaire (Vet-DRQ): five personal resource scales (Self-efficacy (SE), Reflective behaviour (REF), Optimism (OPT), Proactive behaviour (PRO) and Thoughtfulness (THO)), and six job resource scales (Decision latitude (DL), Decision authority (DA), Skills discretion (SD), Support from colleagues (STC), Support from supervisor (STS), Feedback from work (FB)) [30]. The Vet-DRQ is a questionnaire tailored to and validated for the veterinary profession, that can be used for measuring various job demands, job resources, and personal resources. The Vet-DRQ consists of 22 scales. Alpha coefficients of all scales ≥ .70. Because completing the Vet-DRQ is rather time consuming we opted for shortening the scales. Items were chosen on the base of face validity. Responses on a five-point scale were used for all job resources scales (1 = never; 5 = always) and for all personal resources scales (1 = I totally disagree; 5 = I totally agree).

*Work engagement* (WE) was measured using the nine-item version (seven-point scale: 1 = never, 7 = always) of the Utrecht Work Engagement Scale (UWES) [31], with work engagement as a one-dimensional construct and high scores indicating strong engagement.

*Background information* consisted of demographic and occupational details: age, gender, number of years since graduation, number of years of work experience.

### Analysis

The mean scores of personal and job resources and work engagement at Time 1 were compared with those at Time 2 using ‘paired samples T-tests’ in SPSS version 17.0 (SPSS Inc.). Effect sizes were calculated using Cohen’s d [32].

#### Qualitative study

Additional to the quantitative data collection, we conducted a qualitative study comprising individual semi-structured interviews with participants of the development programme. The main objective of the interviews was to find an answer to research question two (how did the development programme affects participant’s personal resources?) and three (how did personal resources affect participant’s work and work environment and participant’s work engagement?). In contrast to group interviews that have a public nature, individual interviews allow to delve deeper into personal experiences [33].

### Procedure

The interviews were conducted 6 months after completing the programme, allowing for the long-term effects to be taken into account. The interviews were semi-structured which means that initial questions were formulated. After the initial response of the interviewee, the interviewer asked new questions for clarification or to encourage the interviewee to supplement the response. The questions were prepared on the basis of the JD-R model. The interviews were conducted by the first author (NM) and lasted approximately 1 h. All interviews were audio recorded.

### Participants

The 16 participants for the interviews were randomly chosen out of the participants who completed the first questionnaire. We sought to interview five persons per cohort. Depending on the number of participants who completed the first questionnaire in each cohort, we approached every second or third person on the list with the question of whether he or she consented to the taking of an interview. No invitee refused. The average age was 29 years (SD 2.9). Two out of sixteen interviewees were male.

### Analysis

With the aim to increase internal reliability [34], transcriptions of the recorded interviews were independently analysed by two of the authors (NM and PvB) by use of deductive, thematic analysis at a semantic level [35]. This approach of the data is driven by theory, in this case the assumptions and a priori themes of the JD-R model (i.e. job demands and resources, personal resources and work engagement). Following the guidelines from King and Horrocks, the analysis of the data is done in three stages [34]. After familiarizing themselves with the data by reading an interview as a whole, both researchers independently started coding the data firstly by identifying codes that refer to personal aspects, secondly identifying codes referring to aspects of the job or the work environment and ultimately identifying codes referring to work engagement (descriptive coding). In the next phase the researchers sorted the different codes into potential themes and collated relevant coded data within the identified themes. The potential themes were then reviewed and discussed till agreement was reached about the themes, their meaning (interpretive coding) and how they fit together (overarching themes), where after they were named. Finally, quotes were selected to illustrate the themes.

#### Ethical considerations

At the time this study was set up, educational research was exempt from institutional board review by Dutch law [36]. It was designed to meet the Helsinki Declaration guidelines [37–39]. In the quantitative study confidentiality of the data was explained in the letter inviting the veterinarians to take part in the survey. We explicitly stated that participation in the study was voluntary and that no one else but the first researcher had access to the raw data. For the qualitative study all interviewees gave written informed consent in response to a letter that explicitly stated that participation in the study was voluntary and that gave assurance of full confidentiality of the data. We adhered to the RATS guidelines for qualitative research [40]. A COREQ checklist is included as an additional file ( Additional file 1).

## Results

### Quantitative study

#### Participants

Participants and non-participants of the programme did not differ significantly concerning the mean levels of job and personal resources and work engagement at Time 1.

### Descriptive statistics

Table 1 shows intercorrelations and the internal consistencies (Cronbach’s alpha) of the variables included in the analysis.

**Table 1:** Intercorrelations between the study variables and Internal Consistencies (Standardized Alpha’s-on the diagonal)

	N	1	2	3	4	5	6	7	8	9	10	11	12	13	14	15	16	17	18	19	20	21	22	23	24
1. DL T1	21	(.77)																							
2. DA T1	21	.708^**^	(.86)																						
3. SD T1	21	−.019	.177	(−.01)																					
4. STC T1	21	.218	.481^*^	.338	(.86)																				
5. STS T1	21	.122	.339	.237	.428	(.91)																			
6. FB T1	21	−.198	−.160	.174	−.045	.487^*^	(.70)																		
7. PRO T1	25	.000	−.020	−.208	.111	.173	.121	(.79)																	
8. SE T1	25	.141	.212	−.163	.109	.252	.146	.717^**^	(.84)																
9. OPT T1	25	.439^*^	.403	−.068	.285	.326	.140	.631^**^	.613^**^	(.83)															
10. REF T1	25	.034	.053	.016	.141	.418	.293	.569^**^	.527^**^	.711^**^	(.85)														
11. THO T1	25	−.032	−.121	.046	.190	.072	.303	.458^*^	.426^*^	.339	.265	(−.53)													
12. WE T1	21	.266	.126	.002	.112	.541^*^	.548^*^	.514^*^	.421^*^	.424^*^	.331	.471*	(.94)												
13. DL T2	21	.768^**^	.572^**^	−.010	.059	.243	.051	.188	.377	.533^*^	.126	.169	.300	(.76)											
14. DA T2	21	.548*	.521^*^	−.073	−.007	.095	−.195	.131	.440*	.359	.207	−.036	.074	.719^**^	(.92)										
15. SD T2	21	.328	.285	.521^*^	.092	.118	−.160	−.297	−.054	−.006	.025	−.236	−.090	.088	.373	(.74)									
16. STC T2	21	.180	.151	−.175	.061	.079	−.208	.102	.466^*^	.196	.388	.087	.044	.030	.526^*^	.533^*^	(.69)								
17. STS T2	21	.565^**^	.269	−.291	−.185	.076	.217	.141	.179	.383	.147	.110	.461^*^	.478^*^	.389	.156	.285	(.78)							
18. FB T2	21	.473^*^	.253	.177	−.019	.285	.472^*^	.067	.213	.381	.242	.258	.436^*^	.364	.127	.292	.193	.676^**^	(.73)						
19. PRO T2	25	.182	.058	−.222	.154	−.042	−.082	.675^**^	.410^*^	.557^**^	.334	.624^**^	.436^*^	.311	.220	−.200	.134	.318	.049	(.86)					
20. SE T2	25	.319	.228	.132	.449*	.195	−.061	.258	.250	.028	−.049	.291	.221	.069	.002	.304	.218	.057	.131	.090	(.53)				
21. OPT T2	25	.306	.087	−.238	.407	.040	−.090	.505^*^	.338	.682^**^	.310	.359	.337	.171	−.063	−.159	.067	.320	.220	.605^**^	.179	(.75)			
22. REF T2	25	−.153	−.055	−.040	.100	.036	.146	.664^**^	.578^**^	.425^*^	.427^*^	.238	.468^*^	.025	.061	−.276	−.038	−.077	−.236	.358	.067	.277	(.75)		
23. THO T2	25	−.229	−.200	.063	−.064	−.051	.039	.555^**^	.265	.151	.235	.482^*^	.273	−.026	−.204	−.251	−.228	−.098	−.221	.441^*^	.247	.099	.551^**^	(.77)	
24. WE T2	21	.376	.220	−.078	−.148	.192	.252	.460^*^	.530^*^	.523^*^	.556^**^	.199	.514^*^	.292	.422	.440^*^	.564^**^	.668^**^	.593^**^	.319	.266	.198	.257	.112	(.90)

*T1* Time 1, *T2* Time 2, *p ≤ .05. **p ≤ .01

#### Are self-reported levels of personal resources increased at the end of the intervention as opposed to their levels at the start of the intervention?

In order to investigate this question, a paired-sampled *t-*test was used for five personal resources and six job resources. As can be seen in Table 2, a significant difference between Time 1 and Time 2 was found in participants for Proactive behaviour (Effect Size (ES) =−0.4, Self-efficacy (ES =−0.6) and Reflective behaviour (ES =−0.6). Results of the control group were not significant, although some moderate effect sizes were found.

**Table 2:** Paired sample *t*-test results for personal and job resources, and work engagement on Time 1 (T1) and Time 2 (T2) in participants and non-participants (Standard deviations in brackets)

	Participants				Non-participants			
	N	Means at T1	Means at T2	Effect size	N	Means at T1	Means at T2	Effect size
Personal resources								
Proactive behaviour	25	2.89 (.67)	3.18 (.63)	−0.4*	10	2.83(.53)	3.15 (.64)	−0.5
Selfefficacy	25	3.37(.79)	3.76 (.43)	−0.6*	10	3.73(.34)	3.83 (.39)	−0.3
Optimism	25	3.68 (.85)	3.92(.74)	−0.3	10	4.05(.54)	3.92(.54)	0.2
Reflective behaviour	25	3.51(.82)	3.88(.41)	−0.6*	10	3.67(.63)	3.77(.52)	−0.2
Thoughtfulness	25	4.14(.40)	4.18(.63)	−0.1	10	3.95(.50)	4.15(.34)	−0.5
Job resources								
Decision latitude	21	2.67(.66)	2.68(.70)	0.0	9	2.96(.72)	2.81(1.03)	0.2
Decision authority	21	3.00(.69)	3.16(.79)	−0.2	9	3.15(.87)	3.52(.50)	−0.5
Skills discretion	21	3.52 (.45)	3.46(.59)	0.1	9	3.59(.60)	3.52(.75)	0.1
Support colleague	21	4.12(.55)	3.99(.60)	0.1	9	4.17(.35)	4.14(.57)	0.1
Support supervisor	21	3.70(.97)	3.71(.96)	0.0	9	4.04(.59)	4.04(.73)	0.0
Feedback from work	21	2.81(.55)	2.76(.73)	0.1	9	2.70(.35)	2.93(.62)	−0.5
Work engagement	21	4.48(1.06)	4.61(.85)	−0.1	9	4.99(.56)	4.89(.83)	0.1

*p < .05

### Qualitative study

#### Summary of results

The important themes that emerged from the interviews seem to be the results of a reflection process that took place over the course of the development programme. The programme resulted in participants reporting increased personal resources such as self-acceptance and self-esteem (personal resources that we did not measure in the online survey), increase of proactive behaviour and increased self-efficacy. Subsequently they reported an increase of perceived job resources and a decrease of perceived job demands. Most participants mentioned that their work engagement was unchanged or increased. Results have been illustrated by quotes (Table 3).

**Table 3:** Quotes from interviews. Capitals between brackets correspond to capitals in text. Numbers between brackets refer to participants in the study

	Quotes
A	“Swapping experiences with others and hearing their stories was very fruitful, how they, experienced their first years as vets, so to speak. Realizing that everybody has ups and downs and you aren’t the only one going around with uncertainties. I was thinking. well, it should get better every year, until you can do everything.” (1)
B	“Being a perfectionist is, like, not being allowed to make mistakes. That was more when I started work. Then you had the idea, my goodness, if I do this, then that happens and the animal dies, the client will be mad at me and then I’m a worthless vet.”(10)
	“And I’ve learned to recognize my own thinking patterns and if I get into a negative spiral I’ve learned to recognize it and to get myself out of it. Or at least, I know roughly how to get out of it. I don’t know if I’ll get out of it but at least I can see when I am in it.”(5)
C	“As well, because I tell myself, OK, I do my work, I do my very best, I do it as well as I possibly can, so it’s less stressful if I make a wrong decision according to the owner, because I can still tell myself that I thought that was the best decision at the time and that’s why I made it.“(1)
D	“Yeah. like, especially that your own opinion counts and that if somebody has a big mouth and always shuts you down, it doesn’t mean that’s the truth, but you can say, OK, wait a minute, I think differently and that you dare to say it. You can just say calmly I don’t agree and this is why I don’t agree and it doesn’t have to lead to a conflict. Partly competence and partly realizing that my opinion counts too.” (4)
E	Refusing a request: “I realized that refusing a request is a good option and it’s OK. And that has directly to do with feeling guilty towards colleagues. At first I felt bad for saying no and now I know that if I don’t say no, I’ll have a bad day and that I’ll be communicating that to colleagues and clients who come in.” (2)
	Taking responsibility: “It’s all about making sure you’ve got things sorted and that you act from a certain conviction. In any case, that you have influence on you own life, it’s not something that just happens, you can choose to say yes, no, OK, maybe, I do it this way or that way.” (4)
F	“….then I think that this has helped me to think more often, it will turn out better than I expect or it’ll be.” (8)
	“When you see that it works it gives you more self-confidence. That is the outcome of it.” (16)
G	Decision authority: “With meetings and that, I usually said nothing because everybody had something to say and I thought, fine. Now I have a say.”(10)
	Communication at an earlier stage: “Now, if I’m bothered about something, I’ll put it forward. At first I didn’t do that at all and then I thought it’s probably part and parcel of the job.” (1)
	Improved work-life balance: “Yes. I can distance myself better from my work, still concerned with your patients and your work but being able to close the door, so to speak, and leave it behind.” (14)
	Actively coping (with) high workload: “So, a whole lot of tasks which are given to me and I have to decide what has priority and what can wait. I used to get stressed out and now I can delegate better, I can say ‘no’ more easily to things. I can say I’ll do that in a bit but I’m finishing this first. Without feeling guilty about it. Eventually you find a way of communicating that to your colleagues without making them feel uncomfortable.“(2)
H	Show more leadership: “And I’ve learned that I must phrase my questions differently, that I should say. I’ve got such and such a patient and I want to do this or that, what would you do? That I first say what I want to do and only then ask what they want to do.”(1)
	Making use of decision authority: “….. that we all have a discussion before making a decision and that I want to be involved in it. Yes, I like the feeling of involvement because it motivates my work.” (13)
I	“What the development programme has done is that, because you think about ‘how do I want my work’ and ‘how do I want my private life’, you get a broader perspective, so to say. The point is, you think “I’m a vet and that’s all”, and then it's rather limited. And when your perspective broadens, you realize there is much more you can do.”(14)

#### How did personal resources increase during the development programme?

### Self-acceptance and self-esteem

Through sharing of experiences, corresponding feelings and thoughts, in the context of the peer coaching meetings, participants recognized that they were not alone in their uncertainty and in doubts concerning their abilities or learning capacities. This allowed a different perspective on the personal situation (A). They became aware of their own thoughts, the areas of tension, and of having a choice whether or not to allow limiting factors to determine their behaviour (B). By analysing their own thoughts they realized that these thoughts caused a great deal of stress and they then learned to replace these by more constructive thoughts (C). The reflection process resulted in participants reporting increased self-acceptance and self-esteem. Participants mentioned that they felt less stressed (D).

### Proactive behaviour

Participants became aware that they always have a choice in acting and learned that they can affect situations (3, 4, 12). They learned to take responsibility for their way of working and living (2, 8, 9, 15) and what prevents them achieving the desired situation or from realizing their stronger sides (7). They learned to cope actively, be proactive and use their influence with the aim to make change happen.

### Self-efficacy and optimism

Training of specific skills in the course of the development programme supported participants in actually deploying new behaviour. Examples of learned skills were ‘giving constructive feedback’, ‘refusing a request’, ‘engaging in conflicts’, ‘leading a conversation or chair a meeting’ (E). When new behaviour was carried out successfully, it strengthened their belief in their personal efficacy and their optimism. This helps the participants to break away from old patterns and fosters their optimism (F).

#### How did these personal resources affect participants’ work and work environment and participants’ work engagement?

### Perceived effects of increased self-conscientiousness and increased self-esteem

According to the participants, the reflection process, of which increased self-consciousness, an awareness of own needs and increased self-esteem resulted, had various effects on job resources and job demands: they mentioned that their communication with clients and colleagues improved, they communicated more and at an earlier stage (1, 4), they perceived increased support from their supervisor (5, 10) and experienced improved relationships with colleagues (5, 7) and clients (11). They made more use of decision authority afforded by their employer (4, 5, 10), and their work-life balance improved by setting limits to workload or by prioritizing tasks (2, 4, 10, 14, 13). Sometimes the reflection process made them aware that their job did not (yet) fit their needs (1, 11, 13) or indeed fitted their needs and competencies very well (7, 3) (G).

### Perceived effects of proactive behaviour

The effect of increased awareness of one’s own influence and consequently developing a proactive approach to work and work environment is also noticeable with respect to job demands as well as job resources. Participants experience an improved work-life balance through active coping with high workload (1, 2, 3, 4, 9, 12, 15) and actively engage in conflicts when necessary (4, 15). Participants also state that they make better use of decision authority (4) or ask for more involvement in practical affairs (2, 10, 13), they seek more feedback (5), give unsolicited feedback when necessary and show more leadership. According to participants this was recognized by clients and colleagues and often led to greater appreciation and support (9). Participants noted that commitment from the employer was a necessary condition to be able to bring about change (16) (H).

### Perceived effects of increased self-efficacy

Increased self-efficacy leads to positive feelings like comfort and satisfaction. Due to these positive feelings participants engage in other extra-role activities or in job design in such a way that the job better fits their individual abilities and preferences. In some cases the increased self-efficacy beliefs encouraged participants to search for another job that better fitted their needs and abilities (11, 5) (I).

### Perceived effects on work engagement

With regard to work engagement participants mention that levels of work engagement differ from day to day and completion of a survey on work engagement is just instant recording (1, 16). For some of them the development programme had no effect on their work engagement (10, 12). According to other participants, engagement increased through insight into their drives, their capabilities and their opportunities (7, 13, 14). Other participants mentioned that an increase of job resources (better communication with colleagues, decision authority, and skills discretion) improved work engagement (2, 4, 5).

## Discussion

This study served three aims. The first aim was to evaluate the effects of a development programme for young veterinary professionals on their personal resources, job resources and work engagement. The second aim was to gain insight into how the programme affected participant’s personal resources. The third aim was to increase our understanding of how these personal resources affected participants’ work and work environment and participants’ work engagement.

### The programme’s effect on participants’ personal and job resources and work engagement

Self-reported ratings of reflective behaviour, proactive behaviour and self-efficacy are significantly increased after the programme as opposed to their level prior to the programme. With regard to the increase of reflective behaviour and self-efficacy one might assume that this might indeed be attributed to the development programme, the more so because these results are supported by the results of the qualitative part of this study. The interviews provided information that made us understand what exactly the intervention did with the participants. They learned to reflect, and through reflection their self-acceptance increased and their thoughts about their own role in the processed changed. As these were the aspects that were aimed to train during the intervention, these qualitative data justify the mechanism through which the intervention worked and resulted in increased personal resources (i.e. proactive behaviour and self-efficacy). It is also in line with previous research that reveals that reflective behaviour is promoted through interactions and peer group meetings [41–45]. The multiple modules approach might have been very helpful again in building self-efficacy. The modules provide different sources of influence that can build participant’s efficacy beliefs [46].

The personal resources ‘optimism’ did not increase. An explanation for this could be that optimism was assessed with a four-item scale that was designed to assess the personality-trait optimism [47]. Respondents were asked to indicate their degree of general agreement “over the past year” with statements such as “I’m always optimistic about my future”. A trait appears to be less developable than a state. This could be an explanation for the fact that optimism did not increase in this study. Future research could pay attention to and distinguish between these two aspects of optimism for example by measuring optimism over the last year and over a shorter period i.e. a week.

The addition of a control group allows us to control for the natural maturation of participants over time and for selection effects. The increase of personal resources in the control group were not significant, although some moderate effect sizes were found for proactive behaviour and thoughtfulness. Participants of the development programme enrolled for the programme voluntarily. Participants of the control group did not,. Therefore these two groups are not fully comparable and it remains unclear whether the people who participated in the programme would have showed the same development as a result of natural maturation as the control group, if they had not been participating in the development programme. The results of the qualitative study however support the assumption that there is a causal relationship between the increase of participants’ personal resources and their participation in the development programme.

Job resources did not increase significantly during the programme, according to our quantitative data. This is not in line with the results from the qualitative part of the study. One explanation could be that each person worked on an own job resource and thus the overall level of job resources did not increase, but it did so for specific resources which differed per participant. An extension of this study with more participants might possibly be able to confirm this hypothesis. Another explanation might be that changing job resources requires support from management that, if not present, may prevent generation of job resources. In a study among newly graduate nurses, healthy work environments improved new graduates transition into professional practice in hospitals [48]. Finally, as the quantitative data are collected immediately after the development programme had finished, while the qualitative data were collected 6 months after the end of the programme, an explanation might be that changes in job resources and work engagement succeed the increase of personal resources and thus require more time to be developed. This is supported by findings of a longitudinal study by Xanthopoulou, Bakker, Demerouti and Schaufeli [24] that suggests that personal resources are related to job resources and work engagement reciprocally and over time. Schaufeli, Bakker and van Rhenen [49] found that changes in job resources were predictive of engagement over a 1 year period.

### How did personal resources increase during the development programme?

The interviews revealed that, in addition to an increase of reflective behaviour, proactive behaviour and self-efficacy, the participants have also developed other important personal resources namely self-acceptance, self-esteem, awareness of own influence and responsibility. The reflection process, which took place in the course of the programme, seemed to be a necessary step for the development of the other personal resources. According to Korthagen [50] the reflection process is a cyclic process and consists of four steps namely: 1) looking back on the action; 2) awareness of the essential aspects; 3) development of alternative methods of action and 4) carrying out (new) planned behaviour. The programme enabled participants to pass through the full cycle of reflection, and hence to develop the above-mentioned personal resources. Apparently reflective skills can be an essential competence in the promotion of a more positive course of the transition period, which takes place in the transition period of young professionals.

The activities during the development programme resulted, among others, in an increased awareness of their own influence on and responsibility for their work and their life: they realized they had a choice. Apparently some participants did not feel that they were in control when entering the programme. Generalized beliefs about control, which concern the extent to which individuals assume they can control outcomes of importance for them, are among those beliefs that influence primary appraisals of situation [51]. As Rotter says “An internal locus of control refers to the conviction that events are contingent upon one’s own behaviour, and an external locus of control refers to the conviction that events are not contingent upon one’s actions but upon luck, chance, fate or powerful others”. These generalized expectancy beliefs have their greatest influence when a situation is ambiguous or new, which is often the case with the young veterinarians. Apparently the programme succeeded in changing beliefs about controllability of events. When self-efficacy levels are high and individuals believe that they can influence their work-environment successfully, job demands are more likely to be perceived as challenging, and job resources as abundant [52].

### How did these personal resources affect participants’ job demands and resources and work engagement?

According to participants of the development programme, the increase in personal resources also gave rise to an increase in job resources. Firstly, the increased self-esteem made participants feel more confident in communicating with clients, colleagues and with their supervisor. This, together with an awareness of their own responsibility to stand up for their own needs and interests, made them search actively for job resources. Whether they succeeded depended also on the work environment and specifically the management of the practice they worked for. “Job crafting may enable employees to fit their job to their personal knowledge, skills and abilities on the one hand and to their preferences and needs on the other hand” [53]. Secondly, the increased self-esteem, gave them another perspective on the existing job resources. For example, thinking that they were worthless veterinarians made them blind to the existing support of colleagues. Their new, more realistic, beliefs about their own fallibilities and capabilities helped them to see that colleagues did appreciate them and were prepared to help them. Thirdly, through increased self-esteem and awareness of their own influence, they took advantage of existing job resources i.e. decision latitude and decision authority for example by taking measures to regulate the workload. The reason that job resources were not increased immediately after completion of the programme may be that it takes some time to change levels of job resources. Another study with a longer term is required in order to confirm this assumption.

With regard to work engagement, differences existed between participants. Some participants mentioned that their work engagement fluctuated daily and related to daily job resources. This is in line with results of Sonnentag [54] and Xanthopoulou, Bakker, Heuven, Demerouti, and Schaufeli [55]. Both studies found that approximately 40 % of the overall variance on work engagement was at the day (i.e. within-individual) level.

### Strengths and weaknesses

Strength of this study is the combination of a quantitative and a qualitative study design. The results of the qualitative study helped us in explaining the results of the quantitative study. For instance, participants mentioned that an increase of personal resources (self-efficacy and pro-active behaviour) stimulated them to search for more job resources in their present job. Increase of job resources thus seemed to follow chronologically the increase of personal resources. It is understandable that it takes more time and a supportive work environment to craft a job. A few limitations of the current study should be mentioned. First, in this study we only used self-report questionnaires and interviews for data collection. Self reports tend to overestimate the effects of coaching interventions [56], and therefore future studies on the effects of such programmes should include other sources for measuring outcomes. An example might be the use of 360° feedback. Second, in order to be able to discriminate between the increase of personal resources as a result of the development programme and an increase of personal resources as a result of one more year practical experience, we compared the results with the results of a control group that did not participate in the development programme. With this goal in mind we could have collected more data on the comparability of the two groups with respect to mental well-being at T1. We did compare mean levels of job and personal resources and mean levels of work engagement. Differences were not significant however this might be because of the small number of respondents in the control group. Through selection bias the control group might have differed on other variables (such as burn-out) than the ones that were included in the survey. In this respect, it seems relevant to gather more information regarding the comparability of both groups concerning well-being, and to try to ensure that both groups are the same size. In order to prevent selection bias, it is important to assign participants randomly to one of both groups, however this will be very difficult as the programme is not free, and applicants participate voluntary in the development programme.

### Practical implications

For educators it is important to know how education or training can contribute to a more positive course of the transition period, which takes place after graduation. Firstly, this study shows that personal resources as reflective behaviour, self-esteem, awareness of own influence and responsibility, proactive behaviour and self-efficacy can be trained. Development of these resources can be initiated by guided reflection with peers. Apparently, it is important that students learn to reflect upon their experiences, their thoughts, feelings and beliefs. Through stimulation of the reflection process, limiting thoughts can be replaced by more constructive (self) beliefs and expectations about the transition period probably can be modified to match reality. By appealing to a proactive attitude and responsibility towards one’s own learning process, educators can stimulate pro-active behaviour and development of positive self-efficacy beliefs. Guiding students in discovering their own needs and core competencies may be helpful to find a job that fits these needs and competencies. It is therefore encouraging that personal development is one of seven competency domains in the veterinary competency framework (VetPro) [57] that plays an important role in the veterinary curriculum in the Netherlands.

Participants noted that commitment from the employer was a necessary condition to be able to bring about change in their working circumstances and/or job resources. We would like to call on employers to invest time and effort into discovering how to create the best conditions for their employees.

## Conclusions

We describe the evaluation of a 1 year multi-modular development programme for recently graduated veterinary professionals. The way this programme is designed enables participants to work on individual learning goals. Through reflection upon experience, feedback and assessment outcomes participants work on the development of individual personal resources. Reflective behaviour appears to be a skill that is necessary to gain insight in the personal needs and for the development of strategies for new behaviour. Participants of the programme perceived increased personal resources 6 months after completion of the programme. We may conclude that personal resources are developable aspects of the self, that can contribute to a positive course of the transition period of young veterinary and probably also other health care professionals.

## Additional file

Additional file 1:
**Consolidated criteria for reporting qualitative research (COREQ-checklist)** [58]. (PDF 395 kb)

## Abbreviations

ES: Effect size
JD-R model: Job demands resources model
RR: Respons rate
SD: Standard deviation
UWES: Utrecht work engagement scale
Vet-DRQ: Veterinary demands and resources questionnaire
VetPro: Veterinary competency framework
SE: Self efficacy
REF: Reflective behaviour
OPT: Optimism
PRO: Proactive behaviour
THO: Thoughtfulness
DL: Decision latitude
DA: Decision authority
SD: Skills discretion
SPC: Support from colleagues
SPS: Support from supervisor
FB: Feedback from work
WE: Work engagement

## Footnotes

1 Both coaches are certified by NOLOC (Netherlands Institute of Career Coaches)
