# Supplementary material for: Effects of a 1 year development programme for recently graduated veterinary professionals on personal and job resources: a combined quantitative and qualitative approach
Source: BMC Vet Res. 2015 Dec 30;11:311. doi: 10.1186/s12917-015-0627-y (PMC4697329; doi:10.1186/s12917-015-0627-y)
Supplement: Additional file 1: — Consolidated criteria for reporting qualitative research (COREQ-checklist) [58]. (PDF 395 kb) [file 12917_2015_627_MOESM1_ESM.pdf]

Consolidated criteria for reporting qualitative research (COREQ): a 32-item checklist for interviews and focus groups

No	Item	Guide questions/description	
Domain 1: Research team and reflexivity			
**Personal Characteristics**			
**1**	Interviewer/facilitator	Which author/s conducted the interview or focus groups	First author NM
**2**	Credentials	What were the researcher's credentials?	DVM, MSc
**3**	Occupation	What was their occupation at the time of the study?	Researcher/ course coordinator
**4**	Gender	Was the researcher male or female?	Female
**5**	Experience and training	What experience or training did the researcher have?	First author has done previous qualitative research projects and has done MSc in psychology
**Relationship with participants**			
**6**	Relationship established	Was a relationship established prior to study commencement?	Former relationship was present with one participant, but otherwise relationship was not established prior to interviews
**7**	Participant knowledge of the interviewer	What did the participants know about the researcher? e.g. personal goals, reasons for doing the research Personal interest in research and reasons for doing it were described prior to the interviews.	They knew nothing about personal interest
**8**	Interviewer characteristics	What characteristics were reported about the interviewer/facilitator? e.g. Bias, assumptions, reasons and interests in the research topic Descriptions of interviewers experience of illness, types of work and approaches	Participants knew that interviewer had been working as a veterinary practioner.

		including references to publicly available written work.	
**Domain 2: study design**			
**Theoretical framework**			
**9**	Methodological orientation and Theory	What methodological orientation was stated to underpin the study?	Transcriptions were analysed by use of deductive thematic analysis
**Participant selection**			
**10**	Sampling	How were participants selected? e.g. purposive, convenience, consecutive, snowball<br>Purposive and snowball	Participants were randomly chosen out of the participants who completed the first questionnaire in the quantitative part of the study. Randomly means that we approached every second or third person on the list
**11**	Method of approach	How were participants approached?	By telephone
**12**	Sample size	How many participants were in the study?	16
**13**	Non-participation	How many people refused to participate or dropped out?	No one has dropped out
**Setting**			
**14**	Setting of data collection	Where was the data collected? e.g. home, clinic, workplace<br>In a place selected by participants. All above options were included.	Most data were collected at participant's home. Two participants visited the workplace of the interviewer Two participants were interviewed at their own workplace
**15**	Presence of non-participants	Was anyone else present besides the participants and researchers?	No
**16**	Description of sample	What are the important characteristics of the Sample?	• 14 female/2 male<br>• Age 24-33<br>• Year of graduation: 2004 - 2009<br>• 14 P working in veterinary practice, 2 P working elsewhere
**Data collection**			

**17**	Interview guide	Were questions, prompts, guides provided by the authors? Was it pilot tested?	There was no pilot testing. Interview approach is described in the methods section
**18**	Repeat interviews	Were repeat interviews carried out?	No
**19**	Audio/visual recording	Did the research use audio or visual recording to collect the data?	Data was audio recorded.
**20**	Field notes	Were field notes made during and/or after the interview or focus group?	Yes
**21**	Duration	What was the duration of the interviews or focus group? Variable. From 50 minutes to 2 interviews of up to 90 minutes.	Approximately one hour
**22**	Data saturation	Was data saturation discussed?	No
**23**	Transcript returned	Were transcripts returned to participants for comment and/or correction?	No
**Domain 3: analysis and findings**			
**Data analysis**			
**24**	Number of data coders	How many data coders coded the data?	Two
**25**	Description of the coding tree	Did authors provide a description of the coding tree?	The approach of the data is driven by theory. Coding is described in the manuscript
**26**	Derivation of themes	Were themes identified in advance or derived from the data?	Categories were identified in advance. Themes were identified after coding of the data
**27**	Software	What software, if applicable, was used to manage the data?	No software was used
**28**	Participant checking	Did participants provide feedback on the findings?	No
**Reporting**			
**29**	Quotations presented	Were participant	Yes

		quotations presented to illustrate the themes / findings?<br>Was each quotation identified?	 <br><br>Yes
**30**	Data and findings consistent	Was there consistency between the data presented and the findings?	Yes
**31**	Clarity of major themes	Were major themes clearly presented in the findings?	Yes
**32**	Clarity of minor themes	Is there a description of diverse cases or discussion of minor themes?	No
